# Social context restructures behavioral syntax in mice

**DOI:** 10.3389/fnbeh.2025.1617091

**Published:** 2025-11-07

**Authors:** Marti Ritter, Hope L. Shipley, Serena Deiana, Bastian Hengerer, Carsten T. Wotjak, Michael Brecht, Amarender R. Bogadhi

**Affiliations:** 1 Department of Biology, Faculty of Life Sciences, Humboldt University of Berlin, Germany; 2 Neuroscience and Mental Health Discovery Research, Boehringer Ingelheim Pharma GmbH & Co. KG, Biberach, Germany; 3 School of Biological Sciences, University of Manchester, Manchester, United Kingdom

**Keywords:** mouse, social behavior, social withdrawal, context, behavioral syntax, behavioral decomposition, behavioral repertoire

## Abstract

**Introduction:**

The study of social behavior in mice has grown increasingly relevant for unraveling associated brain circuits and advancing the development of treatments for psychiatric symptoms involving social withdrawal or social anxiety. However, a data-driven understanding of behavior and its modulation in solitary and social contexts is lacking.

**Methods:**

In this study, we employed motion sequencing (“MoSeq”) to decompose mouse behaviors into discrete units (“syllables”) and investigate whether–and how–the behavioral repertoire differs between solitary and dyadic (social) settings.

**Results:**

Our results reveal that social context significantly modulates a minority (25%) of syllables, containing predominantly stationary and undirected behaviors. Notably, these changes are associated with spatial proximity to another mouse rather than active social contact. Interestingly, a network analysis of syllable transitions shows that context-sensitive syllables exhibit altered network influence, independent of the number of connected syllables, suggesting a regulatory role. Furthermore, syllable composition changes significantly during social contact events with two distinct sequence families governing approach and withdrawal behaviors. However, no unique syllable sequences mapped to specific social interactions.

**Discussion:**

Overall, our findings suggest that a subset of syllables drives contextual behavioral adaptation in female and male mice, potentially facilitating transitions within the broader behavioral repertoire. This highlights the utility of MoSeq in dissecting nuanced, context-dependent behavioral dynamics.

## 1 Introduction

Social interactions are some of the most complex processes across all basic functions required for survival, leading to a high level of specialization of the involved structures and a high cost of dysfunction (Cacioppo et al., 2014; Porcelli et al., 2019). At the same time this high level of complexity leads to an increased vulnerability, e.g., to pathogens or disorders, an example being the occurrence of social withdrawal as a shared feature across major depressive disorder (MDD), Alzheimer’s disease (AD), and schizophrenia (Porcelli et al., 2019). These conditions have a large impact on quality of life and carry a high cost to both affected individual (Chevance et al., 2020) and society, leading to an urgent need for the discovery of treatments (Greenberg et al., 2015; Jia et al., 2018). After previous approaches showed limited success, perspective on translational neuroscience has shifted toward a focus on psychopathology rooted in observable behavior and neurobiological measures (Research Domain Criteria, RDoC), suggesting that focusing on social impairments in complex disorders might lead to an earlier detection and better treatment outcomes (Garner, 2014; Casey et al., 2013; Cuthbert, 2018; Insel et al., 2010).

To facilitate early and effective identification of promising candidate treatments, pre-clinical research makes extensive use of rodent models of disorders found in humans (Bolivar et al., 2007). This approach can also be used in the search for treatments of social impairments (Luo et al., 2023). Mice possess a complex social behavior, influenced by age, kinship, and sex, among other factors. They utilize ultrasonic vocalizations, pheromones, and body contact as modalities of social interaction (Sangiamo et al., 2020; Ricceri et al., 2007). Recent developments in translational research led to refocusing from constrained tasks at shorter timescales, such as the three-chamber social approach task (Nadler et al., 2004) or tube test to determine the social hierarchy between mice (Fan et al., 2019), toward an open arena layout to enable the mice to show more complex behavioral structures and social interactions (Peleh et al., 2019b; Shemesh et al., 2013; Forkosh et al., 2019). But the complexity of social behavior also required agreed-upon definitions for specific interaction motifs to achieve quantifiable and reproducible descriptions.

At its inception, traditional ethological research was limited to qualitative, higher-level descriptions to characterize complex behaviors (Anderson and Perona, 2014). This required a reduced set of dimensions to make behaviors observable (Gomez-Marin et al., 2014), such as focusing on vocalizations or infra-red beam breaks. Even though some of the earliest descriptions of mouse behavior contained “ethograms” matching complex and specific behaviors (van Abeelen, 1964), it was not possible at that time to capture the full complexity of behavior into an ethogram in a data-driven, quantifiable manner. As this limited the possibilities of the neuroscience community to investigate unrestrained, naturalistic behavior, an urgent goal was the development of appropriate tools (Datta et al., 2019). The advent of convolutional neural networks and marker-less pose tracking tools, such as DeepLabCut (Lauer et al., 2022) or SLEAP (Pereira et al., 2022), allowed for the automatic extraction of key body points capturing much of the pose information available in video recordings collected by one or more cameras (Ebbesen and Froemke, 2022), for single (Pereira et al., 2019; Mathis et al., 2018) or multiple animals (Romero-Ferrero et al., 2019; Bewley et al., 2016). In parallel, other approaches to behavior tracking and decomposition arose (Wiltschko et al., 2015), which utilized the recent advances in machine learning and computation (Dennis et al., 2021). The behavioral decomposition approach, which allows identification of recurring short segments of behavior (“syllables”) that form the basis of complex behavior, was effective in characterizing behavioral fingerprints of drugs in disorders, such as epilepsy (Gschwind et al., 2023), using both three dimensional recordings of single mice (Wiltschko et al., 2020) and utilizing body point representations of single or multiple mice (Hsu and Yttri, 2021; Luxem et al., 2022).

A multitude of tools that focus on automated tracking and annotation of social interactions in mice can be found in recent literature (Peleh et al., 2019a; de Chaumont et al., 2012, 2018; Giancardo et al., 2013), including open-source recording setups. Despite advances toward an integration of ethological and comparative psychological approaches (Bordes et al., 2023) most of these approaches are based on parametric definitions of specific behaviors, as in supervised tools (Goodwin et al., 2024), and do not leverage potential advantages of unsupervised behavior classification or standardized benchmarking (Choi and Kumar, 2024). For example, use of motion sequencing in pain quantification (Jhumka and Abdus-Saboor, 2022) and evaluation of behavioral effects of analgesics (Bohic et al., 2023) revealed unique insights into the difference between baseline and analgesia induced behaviors, as well as common global markers of ongoing pain, such as specific pause and grooming modules.

To investigate if an unsupervised behavioral decomposition approach yields insights into the structure of social behavior in mice, we applied Keypoint-MoSeq to videos of mice in dyadic and solitary contexts. By comparing syllable frequency and structure in dyadic and solitary contexts, we show a significant modulation in a subset of stationary, undirected syllables that are not fully dependent on contact with a conspecific. Instead, these modulated syllables play a key role in transitions between syllables as the behavior becomes more diverse in a dyadic context. Furthermore, social behaviors (e.g., social approach or leave) defined through parametric thresholds can be represented in syllable space. However, we did not find syllables or syllable sequences that distinctly map onto social behaviors. Our results suggest that syllables or syllable sequences are sensitive markers for the changes in behavior in the presence of a conspecific but may not correspond to specific “social” behaviors occurring in this context.

## 2 Materials and methods

### 2.1 Animals

Twenty female C57BL/6J mice (6 ± 1 weeks old at time of first experiment) were obtained from Charles River Germany. They were housed in groups of two to three, with the other cage-mates not used in this study and kept in a light- and humidity-controlled facility. The mice not used in this study were used in a parallel study.

### 2.2 Open field arena setup

The experiments were run in a 453 mm × 453 mm × 400 mm (Width × Depth × Height) square arena made from clear plexiglass (Supplementary Figure 1). The arena was equipped with dimmable LED strips and an analog camera (768 px × 576 px, 25 Hz). All sides except for the front were enclosed by a wooden frame, with the front being covered with a red plexiglass door, and the floor being removable to facilitate cleaning. During the tests a stable illumination of around 220lux was maintained. The removable floor was cleaned with soap and water, while the walls were cleaned with ethanol.

### 2.3 Treatment

This experiment was part of a series that centered around treatment with a compound that required oral delivery. We decided to apply the McIlVaine buffer vehicle treatment on the same route, to account for adverse effects caused by the oral gavage procedure. In the parallel cohorts McIlVaine buffer was used to dissolve the given compound. While the mice were not previously trained for the oral treatment, this method is well established in our lab to ensure the lowest possible burden for the animals. All animals were orally injected with the vehicle treatment 20 min before their recordings started. All procedures followed the regulations for animal experimentation enforced by the local district administration’s animal welfare commissioner of the state of Baden-Württemberg.

### 2.4 Experimental procedure

**FIGURE 1:**
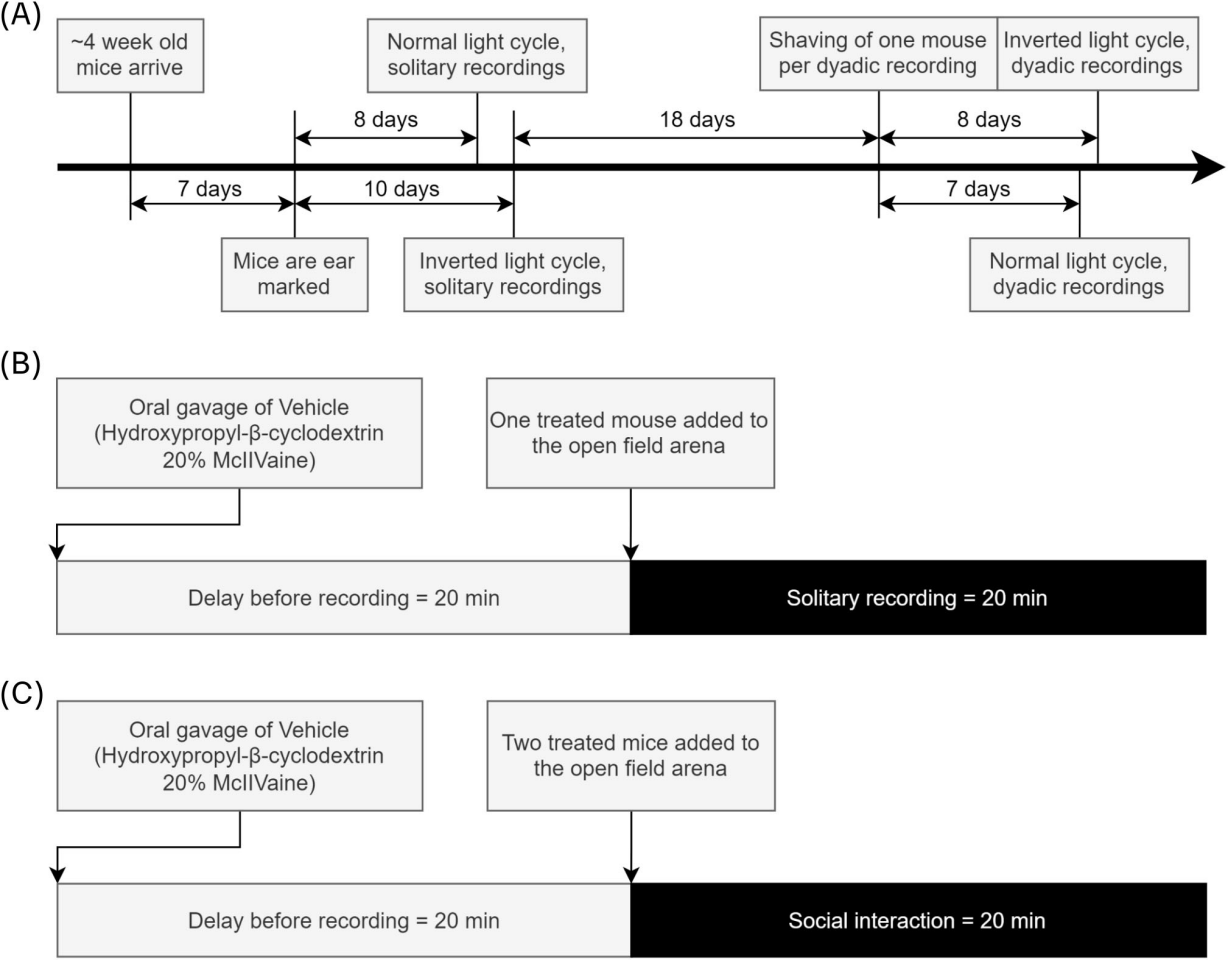
Experiment, procedures, and recordings timeline. **(A)** ∼4-week-old mice were ordered from Charles River and ear marked a week after delivery. Roughly 1 week later the first solitary recordings were performed. More than 2 weeks later one mouse per dyad was shaved, and the dyadic recordings were performed 7 and 8 days later. **(B)** A vehicle treatment was applied orally, and after 20 min had passed the solitary recordings began. **(C)** A vehicle treatment was applied orally to both mice, and after 20 min had passed both mice were placed in the open-field arena and the dyadic recording began.

Half of the mice (*n* = 10) were kept on a regular 12 h light-dark cycle, with the other 10 animals being kept in a reversed 12 h light-dark cycle. This applied to both the solitary as well as the dyadic experiments, with the dyadic experiments occurring between mice with the same light cycle. All experiments began between 8 and 10 am. The timeline of experiments and other procedures is shown in Figure 1A.

#### 2.4.1 Solitary open field test

Mice were orally injected with Hydroxypropyl-β-cyclodextrin 20% McIIVaine and kept in their home cages for 20 min. The oral gavage was performed to consider the impact of treatment stress for future studies on pharmacological treatment. After 20 min had passed, mice were transferred into the recording setups and video-recorded for 1 h. The recordings were started automatically through Stoelting Any-Maze software. Of the 1 h of solitary recordings, only the first 20 min were used for behavioral analysis in this study, to maintain data parity to the dyadic context. The timeline for a single solitary recording is shown in Figure 1B.

#### 2.4.2 Shaved mice

To allow for discrimination between the two mice in each recording, one mouse was shaved (diameter ∼1 cm) 7–8 days before the recording date (see Figure 1A) at a constant location at the upper back behind the head, such that mice could not lick themselves. During shaving the mice were anesthetized with isoflurane and a trained researcher quickly shaved the target location with an electrical hair trimmer. Mice were observed after shaving and if needed, treated with iodine and skin cream to counter any excessive scratching or possible irritation, but we did not observe any issues in the shaved mice.

#### 2.4.3 Dyadic social interaction test

Four weeks after the solitary test recordings, mice were tested in a dyadic social interaction test. Two mice from separate cages were orally injected in quick succession by the same experimenter performing the recording and housed in their home cage for 20 min before starting the dyadic social interaction test. Mice were allowed to freely interact and explore the open field arena for 20 min. The timeline for a single dyadic recording is shown in Figure 1C.

### 2.5 Data collection and analysis

#### 2.5.1 Stoelting any-maze

Stoelting Any-Maze version 7.4 was used to record the videos used in this analysis and to record metadata. No further tracking parameters were extracted from Any-maze for this study. We utilized the built-in TakeNote video observation mode to collect experimenter-scored active and passive contact epochs in the video data.

#### 2.5.2 SLEAP

SLEAP (Pereira et al., 2022) version 1.3.3 was used to track 12 key points on the body of each mouse in the solitary and dyadic video recordings. 4 of the key points were located on the head of the mouse, 4 along the spine, another 2 were located one each at the hips, and 2 further points were labeled along the tail. For the model, we selected a bottom-up design with 800 frames taken from dyadic context and 600 frames taken from solitary context for the training set.

#### 2.5.3 Keypoint-MoSeq

Keypoint-MoSeq (Weinreb et al., 2024) version 0.4.1 was used to extract the behavioral syllables from the keypoints extracted in the previous step. We used 10 of the 12 body points tracked in SLEAP (excluding the tail) to fit the model. The model parameter Kappa was selected iteratively such that the resulting syllable length distribution had its median at 10 frames, representing roughly 400 ms of behavior. The default threshold proposed in the analysis tools provided by the Keypoint-MoSeq Python package was set to 0.5% of total onset proportion and we followed that threshold here.

#### 2.5.4 Validation of syllables

Using the tools provided in the Keypoint-MoSeq package, we named all 32 syllables with a bout proportion above 0.5% of the global distribution. Names and descriptions were chosen before further analysis, to avoid influencing the naming of syllables when results are already known, according to the behaviors shown in the majority of the 24 extracted example videos for each syllable. For a full list and descriptions, see Table 1.

**TABLE 1:** Table of syllables.

Syllable	Label	Bout frequency [%]	Short description
0	dart_forward	6.56	20/24 videos show relatively quick movement from stationary position forward, generally, if possible straight ahead no turns
1	turn_left	5.46	17/24 show left turn, 8/17 related to down from rearing… due to 2D? Usually stationary
2	turn_right_move	5.66	16/24 show right turn, 7/16 related to (down from, sometimes aborted) rearing, same as 1, maybe due to 2D? Compared to 1 this one has after the turn some movement
3	turn_right_stationary	4.82	17/24 show right turn, this time mainly stationary, 11/17 related to moving up into rearing.
4	unclear_contraction	4.28	8/24 show down from rearing, generally seems to have some rightward bias, but not specific
5	movement_forward	4.91	22/24 show general, constant movement forward, usually average velocity
6	sniffing	4.22	23/24 show sniffing, 20/23 related to the corners and edges of the box
7	rearing	3.89	19/24 show movement related to rearing, or “waving” during rearing, mounting… seems to detect more “last” phase of rearing shortly before or during down
8	turn_right_move2	4.23	19/24 usually first light right turn then moving forward, generally long
9	turn_left_short	4.36	21/24 short left turn, sometimes movement afterward
10	sniffing2	3.86	19/24 sniffing again
11	turn_right_stationary2	4.06	20/24 right turn on spot
12	head_raise	3.48	22/24 showing some form of raising head, either on floor from grooming/sniffing up or often related to rearing (14/22)
13	unclear_sniffing	3.28	19/24 some stationary sniffing, but not specific
14	head_raise_left	2.82	19/24 raising head, leftward bias, sometimes weak (17/19)
15	turn_left_with_retract	3.44	19/24 show left turn, most often after short retraction, happens often during down from rearing (leftward) (14/19), but not specific
16	turn_right	3.23	23/24 show right turn, basically the rightward version of 15, also with down from rearing often (16/23)
17	rearing_right	2.55	18/24 show some form of rearing/mounting, mainly rightward (14/18)
18	unclear_sniffing2	2.14	18/24 show some form of sniffing on floor or in the air… maybe rightward bias, but unclear head raise/down
19	unclear_stop	2.16	17/24 show some form of stopping from movement
20	rearing_start	2.14	17/24 show start of rearing some of the rest shows rearing related (down or currently rearing)
21	rearing_left_unclear	1.92	16/24 show some movement turning leftward during a rear
22	sniffing_left	1.93	19/24 show some sniffing at walls while turning their head slightly left and moving sometimes forward
23	rear_down	1.91	17/24 show down from rearing
24	unclear	1.76	No clear behavior
25	sniffing_right	1.3	21/24 show some sniffing, usually against walls, while turning head slightly right… rightward version of 22
26	sniffing_left_turn	1.03	16/24 appear to be sniffing while turning slightly left
27	turn_right_stationary3	1.09	20/24 show some turning right in various contexts
28	turn_right_stationary4	1.2	19/24 show right turn without much other movement
29	movement_unclear	0.97	Appears to be a general movement syllable, maybe slight right turn, but not very specific
30	rearing_sniffing	0.57	17/24 show sniffing while on hindlegs, sometimes with movement forward
31	stop_with_head_down	0.54	16/24 not very clear, but appears to be rather abrupt stop with a short head down movement

Labels and descriptions were determined by using the tools provided with the Keypoint-MoSeq package to create a 24-video overview of the occurrences of each syllable. Behaviors were described according to the behavior shown in most of the videos (see fractions in description column). Labeling was performed prior to the analysis, and minor edits were done for readability.

The example videos that were used to assign these labels are available as Supplementary material. For validation of the content of each syllable we also provide videos specific to solitary and dyadic context.

#### 2.5.5 Body center tracking

**FIGURE 2:**
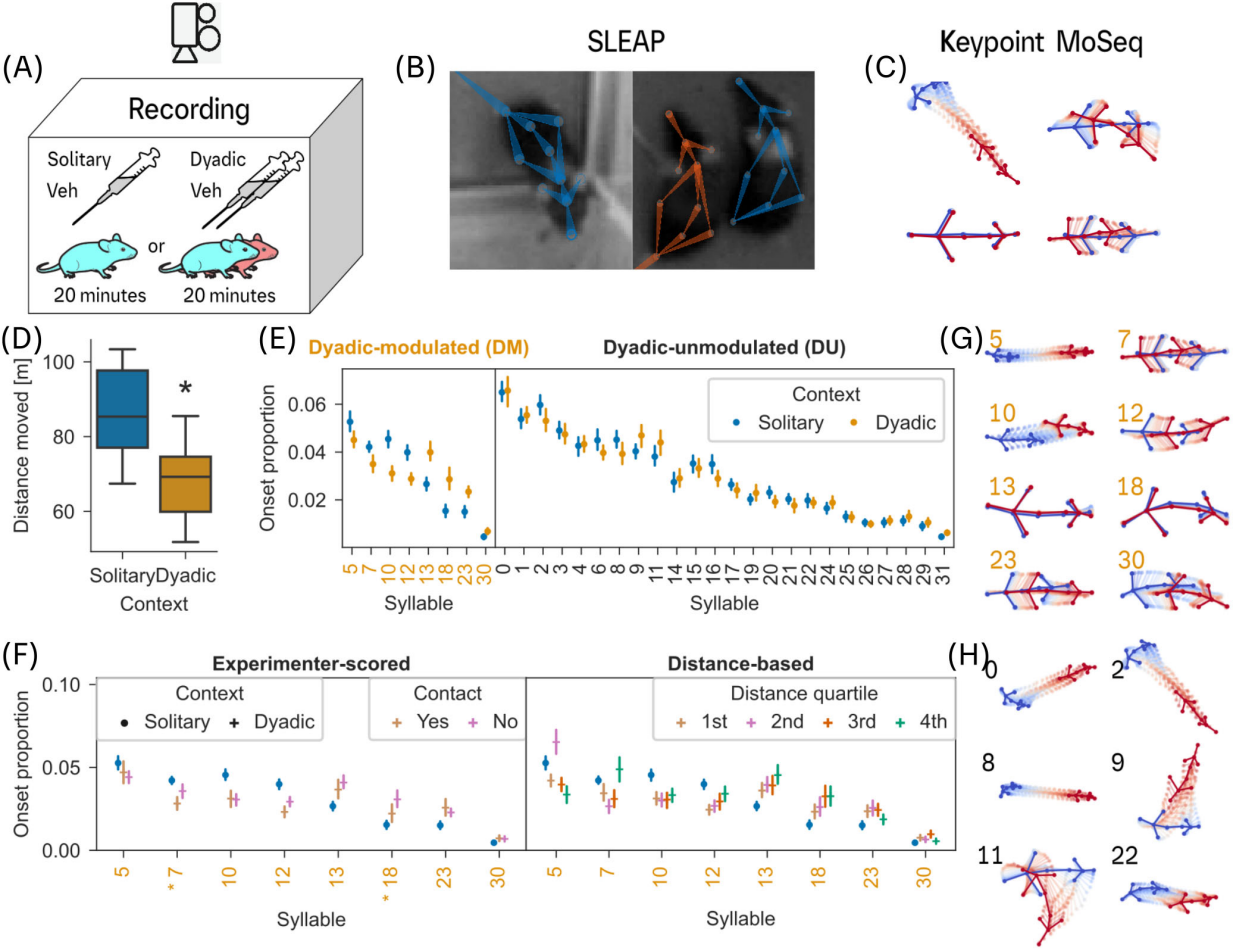
Identification of behavioral syllables in solitary and dyadic contexts. **(A)** C57BL/6J mice were placed into an open field arena, 20 min after an oral vehicle injection, in solitary or dyadic context. **(B)** After video recording in the open field arena, key points on the body and tail were tracked using SLEAP. **(C)** Key points on the body were used to extract behavioral syllables using Keypoint-MoSeq (see section “2 Materials and methods”). **(D)** A comparison of the distances moved in solitary and dyadic context recordings. Asterisk indicates statistical significance (Mann-Whitney U-test, *p* < 0.0001; see section “3 Results”). **(E)** For each of the 32 syllables, a comparison of onset proportions between solitary and dyadic context (Mann-Whitney U-Test, Benjamini-Hochberg correction, *p* < 0.05) reveals 8 significantly modulated (DM; orange colored syllable numbers) and 24 unmodulated syllables (DU). **(F)** Left panel: A comparison of DM syllables’ onset proportion based on its occurrence during or outside of an active contact behavior as scored by an experimenter (see section “2 Materials and methods”). We performed a χ2 proportion test for each syllable’s onset proportion. 2 syllables (7 and 18) show a significant (Bonferroni correction, *p* < 0.05) difference during and outside of an active contact in the dyadic context. Right panel: A comparison of DM syllables’ onset proportion based on the distance between the mice, divided into quartiles, during the syllable occurrence (see section “2 Materials and methods”). For a histogram of inter-mouse distances and the specific quartile distances shown here, see Figure 5B. **(G)** Trajectories of the 8 DM syllables belonging to stationary (7, 12, 13, 18, 23, 30) or non-directional movement (all). **(H)** A selection of DU syllable trajectories with directed (0, 2, 9, 11) and traversal movement (0, 2, 8, 9, 22). In panels **(C,D)**, filled circles and error bars indicate mean and 95% CI respectively. Mouse (Ethan and Kravitz, 2020) and syringe (Bates, 2021) vector graphics adapted from SciDraw.

To explore any differences in mouse behavior, body center tracking was used to calculate the distance moved by a single mouse (Figure 2D), its position (Figure 5A), or inter-mice distance (Figures 5B–E, G). For tracking of body center, we used the mouse centroid calculated in Keypoint-MoSeq by taking the median of all used body points (10 points on the mouse body, excluding the tail). For further information see Supplementary Methods.

#### 2.5.6 Trajectory analysis

We extracted the syllable trajectories from the tracking information collected across all mice. To evaluate similarity between a pair of syllable trajectories, we calculated the cosine distance between position vectors of the pair of syllables. Cosine distance is a measure of distance between two vectors. For further information see Supplementary Methods.

#### 2.5.7 Eigenvector centrality

**FIGURE 3:**
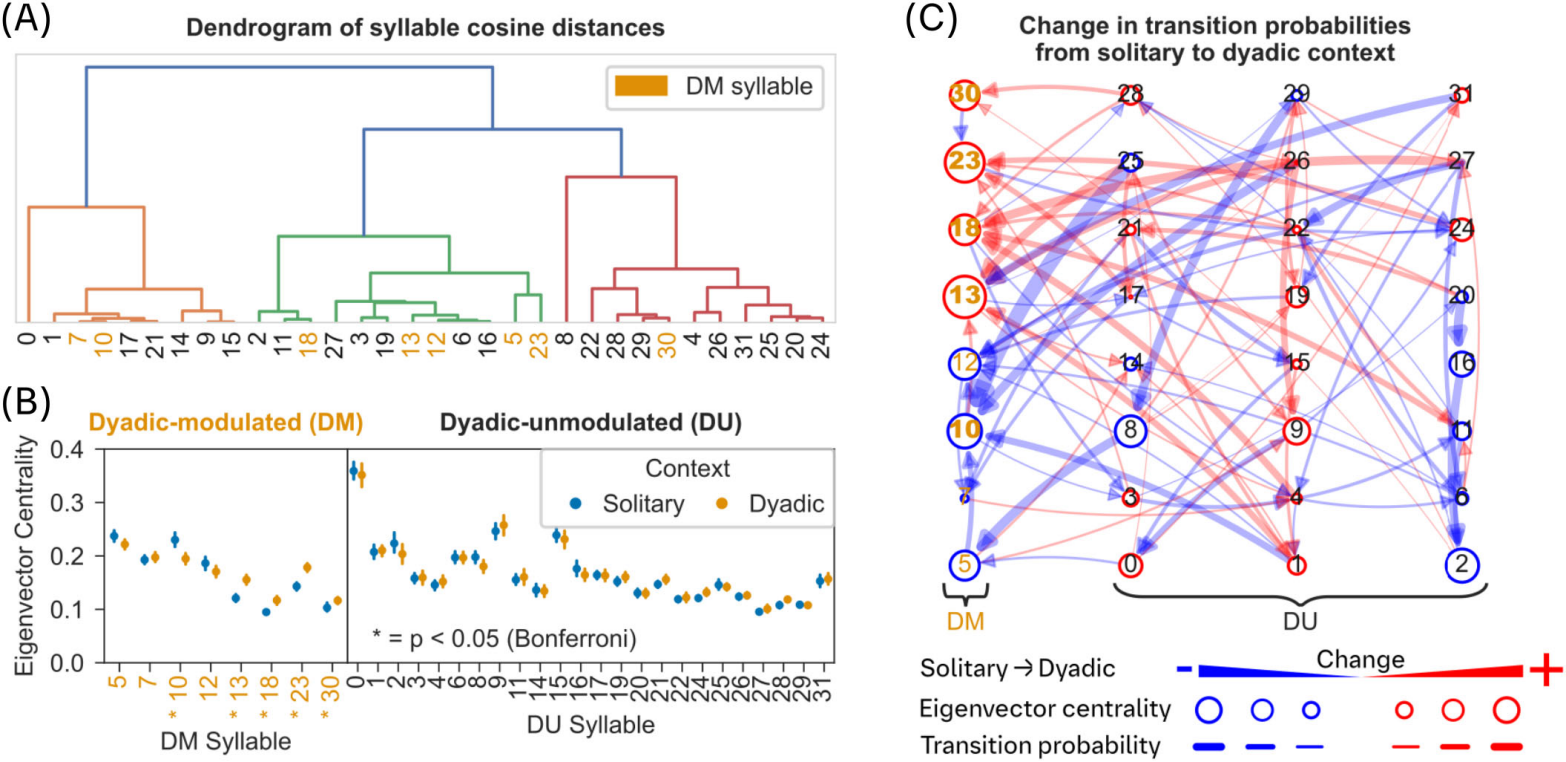
Role of dyadic modulated syllables in the network of syllable transitions during solitary and dyadic contexts. **(A)** The dendrogram of inter-syllable cosine distances shows limited clustering of DM syllables (orange colored syllable numbers). **(B)** A comparison of eigenvector centrality measure between solitary and dyadic contexts (see Methods), based on the network of transition probabilities (outgoing probabilities were normalized to sum 1) between syllables, reveals significant differences for DM syllables (5 out of 8) but not for DU syllables. Asterisk indicates statistical significance (two-sided Mann-Whitney-U test, Bonferroni correction, *p* < 0.05). Filled circles and error bars indicate mean and 95% CI. **(C)** A directed network of syllable transitions modulated by dyadic context with edges indicating transition probabilities between syllable nodes. Edge colors indicate negative (blue) or positive (red) modulation of the transition probability in dyadic context; edge thickness indicates amount of change in transition probability in dyadic context. Syllables are nodes in the network and node diameter corresponds to changes in eigenvector centrality of that node. Syllable nodes with a significant change in eigenvector centrality are indicated in bold syllable numbers [see panel **(B)**].

To identify syllable nodes that may play transitory role in a directed network of syllable transitions, we calculated eigenvector centrality measures for each syllable in the transition network of syllables in solitary and dyadic context (Figure 3B). Eigenvector centrality measure allows for the detection of nodes that are influential in the network, but not necessarily connected to many nodes themselves. For further information see Supplementary Methods.

#### 2.5.8 Social contact scoring

**FIGURE 4:**
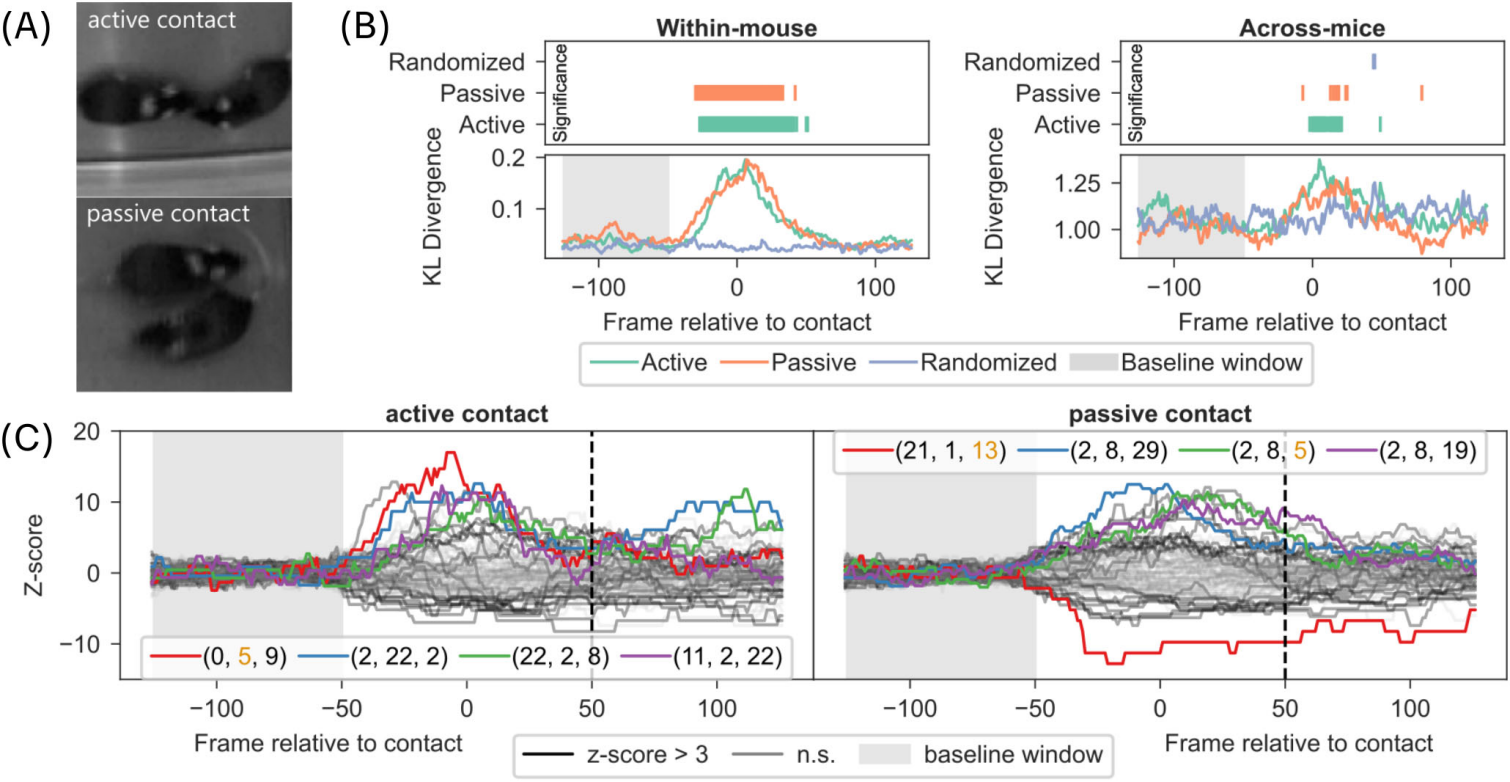
Syllable and syntax composition during experimenter scored active and passive contact behaviors. **(A)** Schematic of an experimenter scoring active and passive contact between mice in the dyadic video recordings. For details on scoring instructions, (see section “2 Materials and methods). **(B)** Left-bottom panel: Time course of difference in frame-wise syllable composition of an individual mice, quantified as DKL, between experimenter-scored behavior and all behaviors is plotted for active and passive behaviors. Left-Top panel: Time course of statistical significance for data in bottom panel compared to shaded baseline window. Right panel: Same as Left panel but using frame-wise syllable composition of two mice instead of a single mouse (see section “2 Materials and methods”). **(C)** The frequency of length 3 syllable syntaxes was aligned to the start of a scored behavior and transformed to z-score using the baseline window (from 100 ms to 50 ms of the start; shaded window). Time course of syntaxes aligned to the start of scored behavior is shown for active contact (left panel) and passive contact (right panel). The top 4 syntaxes are indicated with colored lines and their corresponding syllable composition is shown in legend. Orange colored syllable number indicates DM syllable.

The experimenter was trained in the recognition of social behaviors and the usage of the built-in TakeNote video scoring functionality of Any-Maze and was blind to the light cycle and treatment of mice. Since this study was part of a larger series (see section “2.3 Treatment”), the scorer also scored further videos whose data is not shown here. The scorer classified two main types of behaviors: active social interaction, that is mice engaged in directed interactions, such as touching of the conspecific’s body with the nose and sniffing behavior; passive social interaction was identified as body contacts without active sniffing. If there was uncertainty about how to score a specific segment, the scorer was instructed to mark the segment as both states. The uncertain phases can occur, for example, at the transition between active and passive contact, as the exact moment of transition can be unclear. Around 35% of active contacts and 10% of passive contacts were overlapping in this way. This overlap can also be explained by the input delay on the keyboard used for scoring, and the fact that continuous active contacts do not occur often and last only a few moments, while passive interactions can last multiple seconds. Since we aimed to evaluate the time course of change in syllable and syntax composition during active and passive social contact (see Figures 4B, C), we pooled bouts with less than 6 s duration of the same contact type. This allowed for the application of a 5 s (∼125 frames) pre-contact control window, as shown in Figures 4B, C. We used these pre-contact control windows to ensure that there was a clean onset at the observed bouts, without other bouts of the evaluated behavior occurring in the control window.

#### 2.5.9 Kullback-Leibler divergence

**FIGURE 5:**
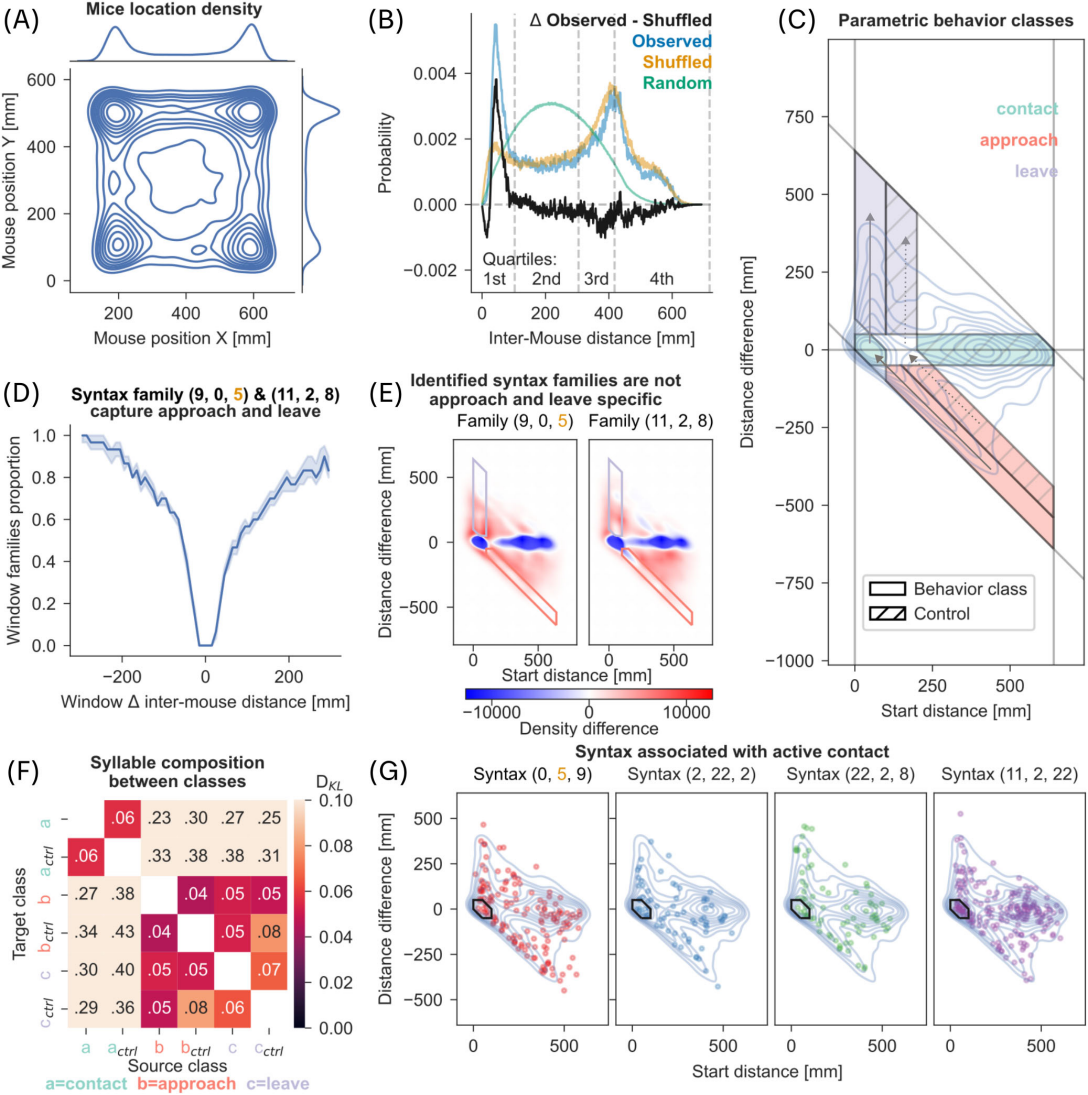
Syntax and parametric behavior classes. **(A)** 2D kernel density estimate of mouse positions sampled from the recordings in dyadic context (2 million data points). Marginal plots show isolated kernel density estimate of each axis. **(B)** Histograms of inter-mouse distances in observed data, shuffled and random control data. Observed refers to inter-mouse distances in recorded data; shuffled refers to inter-mouse distances with the individual positions being shuffled; random refers to inter-mouse distances simulated with randomly selected positions within the arena (10 million point-pairs). The difference of the observed and shuffled histogram is shown in black. Inter-mouse distance quartiles as referenced earlier are marked at the lower end of the plot. **(C)** Parametric behavior classes were derived from observed length 3 syntaxes. Inter-mouse distance (x-axis) and change of that distance (y-axis) were plotted as a kernel density estimate shown as blue contours (KDE, see section “2 Materials and methods”). Based on clusters in the KDE, we defined 3 behavior classes: contact (*x* < 100 mm, x + y < 100 mm), approach (*x* > 100 mm, x + y < 100 mm), and leave (*x* < 100 mm, x + y > 100 mm). Control classes were defined to match change in distance (y-axis) but excluding contact scenarios (see dotted arrows). **(D)** A rolling window (length 30 frames) was used to calculate change in inter-mouse distance (x-axis) and is plotted against the proportion of the window spent in one of the two syntax families (9,0,5) or (11,2,8) (see section “2 Materials and methods”). Line with shaded region represents median and 95% CI. **(E)** KDE with same parameters as in C but limited to one of the two syntax families referred to in panel D. The KDE from panel C was subtracted as baseline. Red areas represent higher density than in C, blue areas with reduced relative density. **(F)** Heatmap of DKL between syllable distributions found in syntaxes belonging to the behavior classes defined in panel C. Darker colors indicate lower DKL. **(G)** Top 4 syntaxes correlated with experimenter-scored active contact, taken from Figure 3C, plotted as scatter on top of the KDE taken from panel C.

To measure differences between distributions of syllables (Figure 4B) and syntaxes (Figure 5F), we applied the Kullback-Leibler divergence (D_*KL*_), or relative entropy. The D_*KL*_ measures the distance between an observed probability distribution and an expected probability distribution. For further information see Supplementary Methods.

#### 2.5.10 Parametric behavior classes

To gain a separation of behavioral syntaxes into parametrically defined behaviors, we used two parameters - inter-mouse distance (IMD) and change in distance (CID) as shown in Figure 5C. The peaks from kernel density plot in Figure 5C were separated into corresponding contact related class and control class (marked with hatched lines) based on IMD. Movement classes (approach and leave) were defined based on CID and IMD. For further information see Supplementary Methods.

#### 2.5.11 Hamming distance

The hamming distance between two sequences is the number of positions that are different in the two sequences. This only includes substitutions, but not rearrangements. In this study, we defined the syntax family as a set of syntaxes with hamming distance <1 relative to the name-giving syntax – for example, for the syntax family (9,0,5), the syntax (9,0,10) would be a member but not (0,5,10). This was used to aggregate the diverse, but highly similar set of syntaxes influencing inter-mouse approaches and leaves as shown in Figure 5D. Further analysis of these families can be found in Figure 5E. For further information see Supplementary Methods.

#### 2.5.12 Principal component analysis (PCA)

**FIGURE 6:**
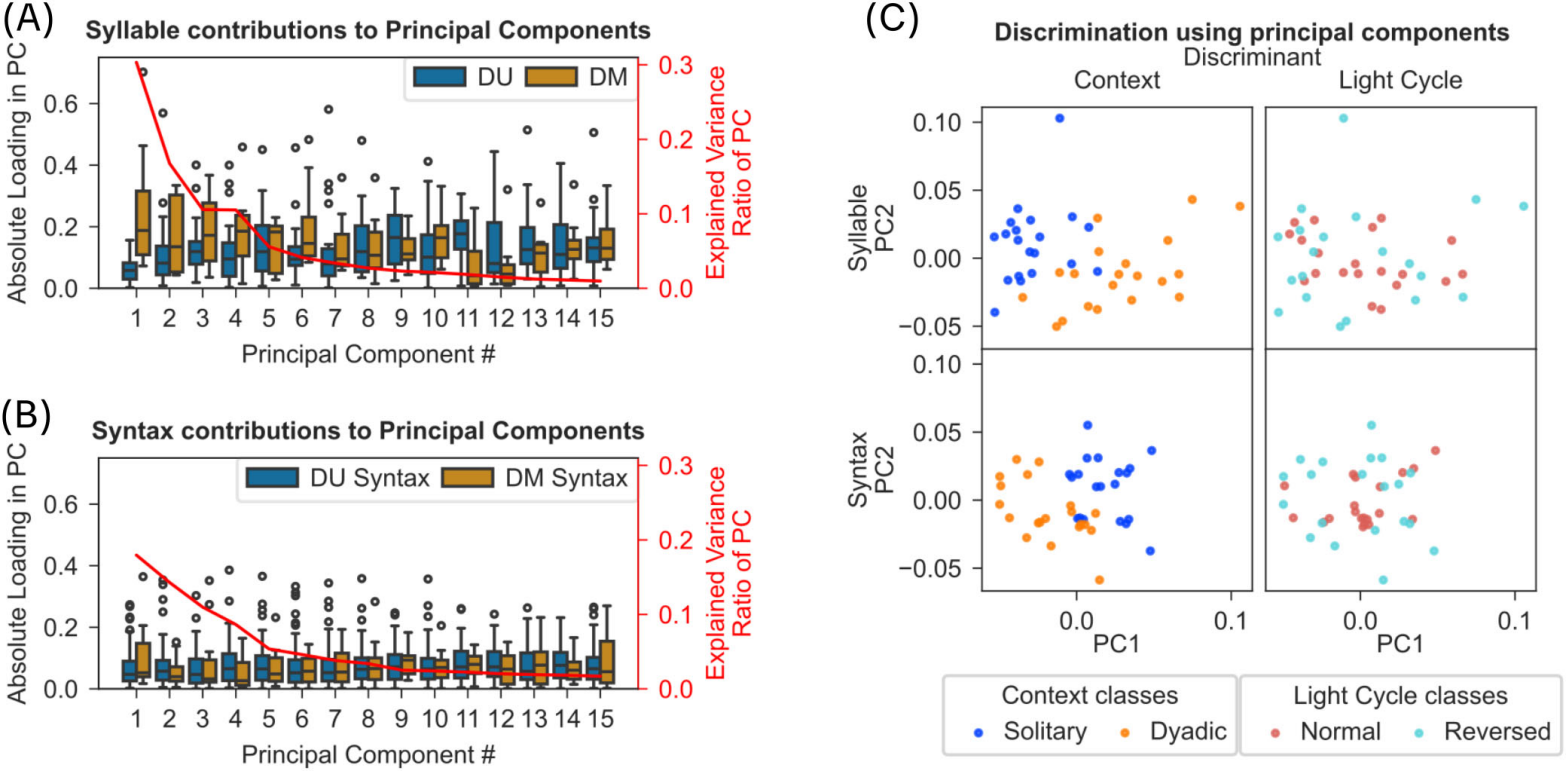
Syllables and syntaxes discriminate solitary and dyadic context but not normal and reverse light-cycle. **(A)** Results from principal component analysis (PCA) of frame-wise occurrences of modulated (DM) and unmodulated (DU) syllables in dyadic and solitary recordings are shown (see section “2 Materials and methods”). The box plots show (with y-axis on the left) the absolute of the loadings on each component for DM and DU syllables. The y-axis on the right shows the percentage variance explained by each of the component. The first 5 PCs explain 74% of the variance and DM syllables contribute significantly more to the loadings of these PCs than DU syllables (one-sided Mann-Whitney U-test, Bonferroni correction, *p* < 0.05). **(B)** Results from PCA on syntax proportions are shown with same conventions as in panel A. DM syntaxes were defined as containing at least 2 DM syllables. **(C)** Scatter plot of the first 2 PCs from syllable-based PCA (top plots) or syntax-based PCA (bottom plots). Colors separate tracks of individual mice in either solitary/dyadic recordings (left plots) or normal/reversed light cycles (right plots).

We used dimensionality reduction with PCA to evaluate if dyadic-modulated (DM) syllables have a larger contribution to the variability across videos (keypoint tracks) of mice in solitary and dyadic contexts (Figure 6A). Similarly, we also evaluated the contribution of DM syntaxes to the variability across videos (keypoint tracks) of mice in solitary and dyadic contexts (Figure 6B). For further information see Supplementary Methods.

### 2.6 Reproduction in separate study

#### 2.6.1 Animals

32 male C57BL/6J mice (7 ± 1 weeks old at time of first experiment) were obtained from Janvier Labs. All animals were injected either orally, subcutaneously, or intraperitoneally with vehicle treatments. Used treatments were NaCl (SC), Na-CMC/Tween-80/NaCl [ml] (IP), and Hydroxypropyl-β-cyclodextrin 20% McIIVaine (PO). All injections were performed 20 min before the recordings started. Along with the treated animals we also used the same number of mice from a different cohort (yet same age, sex, and source) as stimulus animals (see section “2.4 Experimental procedure”). The stimulus animals were kept novel to the treated animals and kept in an isolated facility. All procedures followed the regulations for animal experimentation enforced by the local district administration’s animal welfare commissioner of the state of Baden-Württemberg. Housing and other factors were identical to the primary study.

#### 2.6.2 Experimental procedure

Mice were injected with their corresponding treatment and kept in their home cages for 20 min. After the passing of 20 min the mice were transferred to the recording setups and video-recorded for another 20 min. After the recording of the solitary condition (10 min), a short break (of 20–60 s) occurred, as another (untreated) mouse and a novel object were added to the cubicles contained in the arena. After this, the social condition was recorded (10 min, see Supplementay Figure 2A). The recordings were started and stopped manually through Stoelting Any-Maze software.

## 3 Results

We ran two open field tests (OFT) with video recordings, separated by 4 weeks, on 20 group housed female C57BL/6J mice. The first OFT recording contained an individual mouse in the open arena (referred to as solitary context), and the second OFT recording, performed 4 weeks later, contained two mice from separate cages in the same arena (referred to as dyadic context). In preparation of future pharmacological studies, all mice were orally injected with a vehicle treatment 20 min before the start of the recordings to account for potential adverse effects caused by the oral gavage procedure. Considering potential light cycle effects on behavior, one half of the mice were housed under normal, and the second half were housed under reversed light cycle conditions. However, we could not identify significant differences in syllable expression between the two light cycle groups (see Supplementary Methods; Mann-Whitney U-Test, Benjamini-Hochberg correction, *p* ≤ 0.05) and hence the data were pooled from the two light cycle groups.

We analyzed 20 min of video containing a single mouse in solitary and dyadic contexts using SLEAP (Pereira et al., 2022) to track a set of 12 key body points on each mouse. We fitted a Keypoint-MoSeq model (Weinreb et al., 2024) to a subset of 10 key body points to extract repeated elements of mice behavior, referred to as behavioral syllables (see section “2 Materials and methods”; Figures 2A–C). The output syllables were aggregated by either their onset (bout) proportion, representing the proportion of syllable bouts regardless of individual bout lengths in frames, or frame proportion, representing the total proportion of the recorded frames spent in one syllable. After filtering for total onset proportion, we extracted a total of 32 syllables which were used in subsequent analyses (see Supplementary Figure 3A). Syllables with a bout proportion below the threshold often occurred in clusters (Supplementary Figure 4A) and were observed to relate mostly to self-grooming (Supplementary Figures 4C, D). These clusters occurred in both solitary and dyadic contexts but did not meaningfully differ between the two contexts (Supplementary Figure 4B).

### 3.1 Dyadic context modulates a small subset of MoSeq identified syllables

To investigate the structure of social behavior through behavior decomposition, we first asked if behavioral “syllables” identified through decomposition (Keypoint-MoSeq) are sensitive to modulation in solitary and dyadic contexts and whether this modulation is dependent on physical proximity between the two animals. Before comparing syllables in solitary and dyadic contexts, we verified whether parameters related to movement revealed significant changes in mouse behavior between solitary and dyadic contexts (von Ziegler et al., 2023). By approximating the output of traditional body center tracking (see section “2 Materials and methods”), we detected a significant reduction in distance moved of mice between solitary and dyadic contexts (Mann-Whitney U test, *p*≈1.83e-5; Figure 2D).

A comparison of onset proportion of all 32 syllables in solitary and dyadic contexts revealed a subset of 8 syllables that differed significantly between contexts (Figure 2E; see Supplementary Methods; Mann-Whitney U-Test, Benjamini-Hochberg correction, *p* ≤ 0.05). From here onward, the 8 significantly modulated syllables will be referred to as dyadic-modulated (DM) syllables and the remaining 24 syllables will be referred to as dyadic-unmodulated (DU) syllables.

Because the mice move less in dyadic context (Figure 2D), one may deduce that DM syllables are likely associated with active contact but may not be influenced by physical separation between mice outside of an active contact. To evaluate the effect of physical proximity on DM syllables, we compared the onset proportions of syllables during experimenter-scored active contact (for definition see section “3.2 Modulated syllables drive the syllable transitions in dyadic context”) to no contact. Results from a two-way ANOVA revealed a significant main effect for DM syllables and active contact [F(7, 299) = 49.61, *p* < 1e-45; F(1, 299) = 5.07, *p* = 0.03) with a significant interaction between DM syllables and active contact (F(7, 299) = 2.21, *p* = 0.03). However, a closer inspection with a *post hoc* χ^2^ contingency test for each syllable revealed few significant syllables (2 out of 8, Bonferroni correction, *p* < 0.05). In addition, we checked if the distance between mice is associated with the modulation of the DM syllables by comparing onset frequency in different distance quartile. A two-way ANOVA revealed a significant main effect for DM syllables and distance quartiles [F(7, 603) = 93.62, *p* < 1e-91; F(3, 603) = 3.87, *p* = 0.01] with a significant interaction between DM syllables and distance quartile [F(21, 603) = 8.67, *p* < 1e-22]. These findings reveal that majority of the DM syllables are not directly linked to an active contact but are associated with physical proximity to a conspecific (Figure 2F, left panel; but also Supplementary Figure 3B; specific quantiles: 1*^st^* = 102 mm, 2*^nd^* = 304 mm, 3*^rd^* = 418 mm, 4*^th^* = 652 mm, see Figure 5B for histogram and marked quartiles).

An inspection of the trajectories of all DM syllables reveals high proportion of stationary, and non-directional behaviors, for example rearing as in syllable 7 (Figure 2G). In contrast, most directional behavior (left or right oriented movement as in syllable 9 and 11) falls into the DU group (Figure 2H and Supplementary Figure 3C; one-sided Mann-Whitney U-test, Bonferroni correction, *p* < 0.05). To quantify the differences within and across DM and DU syllables, we used cosine distance to quantify similarity (see section “2 Materials and methods”; Figure 3A). The dendrogram of similarity measure did not reveal any systematic clustering within and across DM and DU syllables. However, using an extended dataset (see section “2 Materials and methods”), we found that most of the DM syllables are similar when compared to DU syllables (Supplementary Figure 5A).

These results show that a subset of behavioral “syllables” identified through decomposition are sensitive to modulation in solitary and dyadic contexts. Surprisingly, most of the modulated syllables, dominated by stationary undirected behaviors, are not linked to an active contact but are associated with physical proximity to a conspecific outside of an active contact.

### 3.2 Modulated syllables drive the syllable transitions in dyadic context

Because there was no association of an active contact with most behavioral syllables in the presence of a conspecific (dyadic context), we next investigated whether there is a distinct role for DM syllables in the transitions between behavioral syllables, potentially suggesting a contribution to complex (consisting of more than one syllable) social behaviors.

We employed eigenvector centrality to quantify the role of DM syllables in the transitions between syllables (see section “2 Materials and methods”). Briefly, eigenvector centrality is a measure of how well a node is connected to nodes which themselves are well connected to individual nodes within a network (Negre et al., 2018). Here, the eigenvector centrality of a syllable is calculated based on the directed graph of transition probabilities. We found a significant change in eigenvector centrality, during the dyadic context, for most of the DM syllables but not for any of the DU syllables (Figure 3B; two-sided Mann-Whitney U-test, Bonferroni correction, *p* ≤ 0.05). This finding reveals a crucial transitory role for DM syllables in the network of syllables defining complex social behavior in the presence of a conspecific (dyadic context).

During the dyadic context, most of the significantly modulated transitions (8/9, two-sided Mann-Whitney U-test, Bonferroni correction, *p* ≤ 0.05) target DM syllables as reflected by eigenvector centrality (see arrows pointing to the first column with DM syllables in Figure 3C). In addition, we observed an overall increase in syllable transitions (Supplementary Figure 5B; one-sided Mann-Whitney U Test, *p* ≤ 0.05) and the strongest changes in transition probabilities (thick lines in Figure 3C) were mostly a result of reduced transition probabilities (blue lines), originating in DU syllables (see Supplementary Figure 5C).

Due to a weak association between contact and DM syllables (Figure 2F), the exact role of these syllables in social interactions is not clear. However, we observed that behavior in general becomes less stereotypical (or structured) when mice are in a dyadic context (see Supplementary Figure 5D). Regardless, these results suggest an important role for DM syllables as “transfer nodes” in the behavioral program to construct a complex socially driven behavior in the presence of a conspecific (dyadic context).

### 3.3 Changes in syllable composition reflect active and passive contact behaviors

Given that DM syllables play an important role of “transfer node” in the behavioral syllable transitions to form complex behavior in dyadic context, we investigated whether complex behaviors, such as active or passive contact behaviors, are captured through changes in the composition of syllables or sequences of syllables, referred to as syntaxes in the following observations.

For this purpose, we tasked a fellow researcher to score active and passive social contacts in the dyadic recordings (see section “2 Materials and methods”). To quantify changes in syllable composition, in an individual mouse during active and passive behaviors, we computed Kullback-Leibler divergence (D_*KL*_), a measure of statistical distance between two distributions, between framewise distribution of syllable proportions in active or passive contacts to an overall distribution of syllable proportions in dyadic context (see section “2 Materials and methods”). The time course of D_*KL*_ reflects changes in syllable composition aligned to start of active or passive behaviors (Left panel in Figure 4B). The results show a significant change in syllable composition (D_*KL*_), in an individual mouse, at the time of active and passive behaviors. We verified that this change is fully attributable to active and passive behaviors since the same analysis on randomized sequence of syllables did not show any change in syllable composition (Figure 4B; z-score see Supplementary Methods).

We next asked if active and passive behaviors also reflect changes in joint syllable composition of two mice by computing framewise Kullback-Leibler divergence (D_*KL*_) between joint distributions of syllable proportions between two mice during active or passive contacts to an overall joint distribution of syllable proportions in dyadic context. The time course of D_*KL*_ shows a weak but continuous and significant change in joint syllable composition aligned to start of active behavior but less so for passive behavior (Right panel in Figure 4B).

To investigate if sequences of syllables (syntaxes) signal active and passive contact behaviors, we extracted length 3 syntaxes (i.e., sequences comprising 3 syllables), as this corresponded to about a second of behavior with a median syllable duration of 10 frames (see section “2 Materials and methods”). A multitude of syntaxes are significantly associated with active (25 syntaxes) and passive (24 syntaxes) contact behaviors (Figure 4C; see Supplementary Methods for statistics). However, there was little overlap of syntaxes between active and passive contact behaviors (Figure 4C, 8 significantly associated syntaxes shared between active and passive). In addition, we observed few DM syllables in the top 4 most significantly associated syntaxes for both contact behaviors. Furthermore, inspection of example trajectories for the two syntaxes most associated with active contact suggests a behavior related to circling around a conspecific (Supplementary Figures 6A, B). Similarly, the syntax most associated with passive contact included mostly stationary behavior, while the second syntax was related to a turning behavior followed by movement (Supplementary Figures 6C, D).

These results show that experimenter defined active and passive contact behaviors are represented in the syllable space and there are a diverse set of syllable sequences that are modulated in contact behaviors. Furthermore, DM syllables are underrepresented in most of the contact-associated syllable sequences, in line with the previously observed modulation of DM syllables regardless of contact (Figure 2F), suggesting their role in aiding transitions between syntaxes actually associated with contact behaviors.

### 3.4 Individual syllable sequences do not map onto specific behaviors

The finding that experimenter defined contact behaviors are represented in syllable and syntax space prompted us to ask whether social behaviors, such as social approach and leave defined in a parametric space (Peleh et al., 2019a), can be mapped onto syllable and syntax space. First, we verified if there is a propensity for targeted behavior in our dataset by calculating distributions of mice positions (Figure 5A) and probability of inter-mouse distance (Figure 5B). Our data shows that mice preferred to position themselves in corners of arena (Figure 5A), and that the co-location of two mice in the same corner was a coordinated event, rather than a random occurrence (Figure 5B).

Before parametrically defining behaviors, we extracted inter-mouse distance during length 3 syntaxes and mapped them onto a space representing the inter-mouse distance at the start of the syntax and change in distance by the end of the syntax (Figure 5C). From the density plot of all syntaxes in this space (blue contours in Figure 5C), we were able to identify four density zones that specify parametrically defined behaviors – targeted approach and leave, as well as stationary behaviors in low distance (contact) and stationary behaviors with high distance. We further defined control zones as those that showed the same relative movements but did not relate to contact between the mice (see dotted arrows and hatched areas in Figure 5C and section “2 Materials and methods”). We choose to use syntaxes rather than syllables since syntax timescales were more apt for the description of targeted long-distance movements and longer stationary behaviors.

For the two distinct behaviors of social approach and leave (Figure 5C), we investigated whether there is a dominant representation by specific syntaxes. We tracked the change in distance over a rolling 30 frame window, matching our definition of length 3 syntaxes. It is evident that nearly all the windows in which inter-mouse distance changed by more than 200 mm were fully represented by either one of two syntax families (see Figure 5D). A syntax family is defined as a set of syntaxes with at most one changed syllable relative to a reference syntax (the family was named after the reference syntax; see Hamming Distance in Methods). We found that social approach and leave were fully represented by two syntax families (syntax 9,0,5 and syntax 11,2,8; see Figure 5D) and both contained a syllable with a long-distance traversal trajectory (syllables 9&0 and 2 respectively, see Figure 2H), suggesting both syntax families capture behavior either targeted toward or away from a conspecific. Inspection of sample trajectories included in these syntax families revealed that one syntax family (9,0,5) was usually associated with a leftward turn followed by a movement straight ahead while the other syntax family (11,2,8) was associated with a rightward turn followed by movement (Supplementary Figures 7A, B). We interpret these syntaxes as corresponding to a mouse reorienting toward or away from a conspecific, during an approach and leave respectively.

We asked if the above finding that social approach and leave were fully represented by two syntax families extended in the opposite direction i.e., whether the two syntax families specifically map onto the social approach and leave behaviors. When we mapped all occurrences of the two syntax families onto parameter space (Figure 5E), we did not identify a direct mapping between a syntax family and a specific behavior class as defined in our parametric space (Supplementary Figure 8A; χ^2^ contingency test, Bonferroni correction, *p* ≤ 0.05, but see Supplementary Methods). Since we could not identify an association between a syntax family and a behavior, we checked if syllable composition differed between behavior classes. We calculated D_*KL*_ on the syllables contained within each syntax, between a pair of behavior classes. We observed no separation between approach and leave classes further confirming that there is not a specific syntax, at least in our dataset, that directly maps onto approach and leave classes. Furthermore, we noted a modest separation between all movement classes (approach and leave) and stationary (contact) classes respectively (Figure 5F, for analysis of syntaxes see Supplementary Figure 8B). However, mapping all occurrences of syntaxes associated with active contact (from Figure 4C) onto our parameter space did not reveal any specific syntax associated with active contact behavior (Figure 5G). We also confirmed that there was no specific syntax associated with passive contact behavior (Supplementary Figure 8C).

These findings show that while mapping of a behavior to syllable space is possible with a one-to-many mapping, we could not find evidence for distinct syllable sequences that map onto specific behaviors.

### 3.5 Syllables allow discrimination of solitary and dyadic behavioral modes

Based on the lack of evidence for specific syntax or syllable descriptors of social behavior, we aimed to find out if syllables or syntax provide discrimination of solitary and dyadic contexts. First, we evaluated the variance captured by syllables and syntaxes using principal component analysis on the proportion of frames containing each syllable for each mouse (see section “2 Materials and methods”). We separated the contributions of dyadic modulated and unmodulated syllables to each of the principal components (Figure 6A). Results reveal that DM syllables capture significantly more variance compared to DU syllables – the first five PCs explaining ∼73% variance have significantly higher DM syllable loadings (one-sided Mann Whitney U-test, *p* < 0.005; see Figure 6A). However, we did not find such a difference for syntaxes (same test, Figure 6B).

Furthermore, using the above identified principal components, we observed a clear separation between solitary and dyadic contexts, while the same approach did not lead to a separation between two light cycle conditions (Figure 6C).

Since we were able to show a clear separation of solitary and dyadic modes using the first 20 min of recordings, we wondered whether this separation persists if we used the solitary segments following the first 20 min (i.e., 20–40 min and 40–60 min). Comparing the difference in onset proportion across all three 20-min segments of solitary recordings to the dyadic context (see Supplementary Methods), similar to the analysis shown in Figure 2E, revealed that the number of significantly modulated DM syllables grew with the time spent in the arena (see asterisk marked syllables in Supplementary Figure 9; Mann-Whitney U-Test, Benjamini-Hochberg correction, *p* ≤ 0.05). The specific syllables varied, but a subset of 4 syllables showed persistent dyadic modulation (syllables 7, 10, 12, and 23). When evaluating eigenvector centrality in the same manner (Mann-Whitney U-Test, Bonferroni correction, *p* ≤ 0.05), only one syllable remained significantly modulated between all solitary segments and the dyadic context (syllable 10).

**FIGURE 7:**
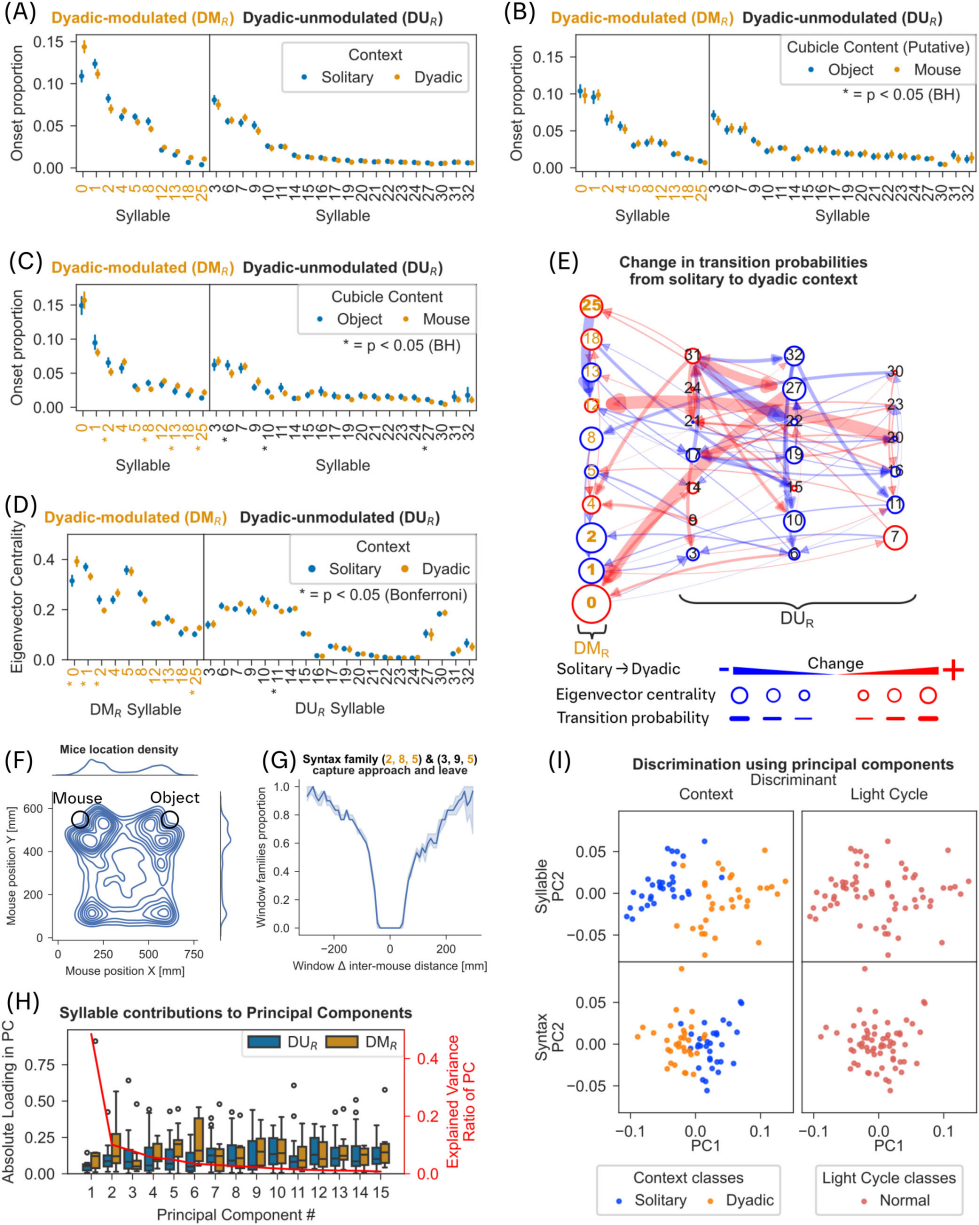
Reproduction of results in a separate study. For methods see Supplementary Figure 2. Where applicable panels apply the same tests and corrections as the reference panel. **(A)** Compare Figure 2E. **(B)** Onset proportion during solitary phase, with colors split for onset proportions observed in contact with later object and mouse cubicles. No significantly different syllables could be found (Mann-Whitney U-Test, Benjamini-Hochberg correction, *p* < 0.05). **(C)** Onset proportion during dyadic phase, again split for now filled object and mouse cubicles. Asterisks denote significantly different syllables (Mann-Whitney U-Test, Benjamini-Hochberg correction, p < 0.05). **(D)** Compare Figure 3B. **(E)** Compare Figure 3C. **(F)** Compare Figure 5A; but see Supplementary Figure 2B. **(G)** Compare Figure 5D. **(H)** Compare Figure 6A. **(I)** Compare Figure 6C.

We were also able to show that the qualitatively same effects as shown here also apply to the solitary and dyadic context in a situation where the conspecific is limited to a cubicle, with male mice treated through subcutaneous and intraperitoneal injection in addition to oral gavage, and with a novel object also present (see Figure 7 and Supplementary Figure 2).

As it was possible that the observed shift in the behavioral repertoire is purely a response to a “novel” stimulus, and not a “social” response, we also compared the behaviors shown around a novel object and a novel conspecific in the second study. We analyzed the syllable onset proportions in the same manner as in Figure 2E (Mann-Whitney U-Test, Benjamini-Hochberg correction, *p* ≤ 0.05).

A set of 10 dyadic modulated syllables was detected and will be referred to as DM_*R*_, to differentiate them from the set of DM syllables described in the other figures. Contact with either cubicle was defined as distances below 10 cm to the center of the cubicle. When both cubicles were empty (solitary phase) we observed no significantly modulated syllables between the two cubicles, as expected (see Figure 7B, cubicles are named according to their later content). During the “dyadic” phase, we observed 7 significantly modulated syllables between object and mouse cubicles, with 4 of these syllables being found among the previously detected DM_*R*_ syllables (see Figure 7C). While there may be a novelty component to the syllable modulations when comparing ‘solitary’ and ‘dyadic’ contexts in that experiment, we can clearly observe significant syllable modulations when comparing syllables around a novel object and a conspecific suggesting that social (dyadic) context modulates the syllable composition in mice even when social context occurs in the vicinity of a novel object context.

These results demonstrate that syllables and syntaxes, particularly DM syllables, provide a sensitive and robust description of behavior to discriminate behavioral states in the presence or absence of a conspecific.

## 4 Discussion and conclusion

A growing number of tools are available to accomplish the data-driven segmentation of the full behavioral repertoire of mice (See reviews: Choi and Kumar, 2024; Datta et al., 2019; von Ziegler et al., 2021). However, it is not known if such an approach, to segment or decompose the behavior into its recurring elements, yields insights into the structure of social behavior in mice. Here, we investigated the structure of social behavior using Keypoint-MoSeq (Weinreb et al., 2024) to videos of mice in a dyadic and solitary context. We found that in the presence of a conspecific, mice moved less and significantly modulated the usage of a small subset of mostly stationary and undirected syllables, referred to as dyadic modulated (DM) syllables (Figures 2D, E; Supplementary Figures 3A, C). Surprisingly, for most of the DM syllables, this modulation in usage was not associated with the occurrence of experimenter-scored contact between the mice (Figure 2F). Instead, DM syllables played a significant role as transitory nodes in the syllable transition network (Figures 3B, C). Importantly, we found significant changes in composition of syllables and syntaxes (length 3 syllable sequence) aligned to experimenter-defined contact behaviors (Figures 4B, C). In addition, we identified 2 syntax families that captured approach and leave behavior as defined through parametric thresholds (Figures 5C, D; Supplementary Figures 7A, B). However, we did not find evidence for distinct syntaxes that map onto specific complex behaviors. Overall, our results show that behavioral syllables can be used as descriptive and sensitive markers to detect changes in behavior in the presence of a conspecific, but specific social behaviors may not map onto individual behavioral syllables or a specific syntax thereof.

A possible caveat of our study could be that the experiments were performed with one set of mice across both contexts. We ran the two recordings (solitary, dyadic) in the same set of open field arenas 4 weeks apart. It is possible that during the second recording the arena may not have been a novel environment for mice. Based on available literature the long-term spatial memory of the arena will be negligible after a span of 4 weeks, as most assessments of spatial memory work with periods of up to 2 weeks after training session over multiple days (Sharma et al., 2010). Similarly, movement patterns of our mice could potentially also evolve during inter-experimental phase (4 weeks) and might have contributed to our findings, but it can be expected that social contact with a conspecific outweighs these effects. The estrous cycle most likely varies across individuals and recordings and might contribute to social preference (Chari et al., 2020). However, as solitary and dyadic context behaviors were clearly distinguishable in our dataset, we conclude that the estrous cycle-related variance must be below the level of behavioral changes in a dyadic context. Furthermore, we were able to show the qualitatively same effects in male mice, with both recordings occurring with only a short break of up to a minute, leading us to assume that the aforementioned factors do not determine the effects described in this study (see Figure 7 and Supplementary Figure 2).

To detect as much of the behavioral repertoire as possible the Keypoint-MoSeq model was fitted to the entire recorded dataset, including full 1 h solitary recordings and 20-min dyadic recordings. Potentially this 3:1 disbalanced dataset could introduce a bias toward behaviors shown in solitary context, but as we treated the solitary state as the baseline, effects of the dyadic context would still be visible in the following analysis. In addition, when analyzing a second study with the same amount of data for both contexts, we were also able to show the qualitatively same effects (see Figure 7). We also limited the analysis of the extracted syllables to 20-min segments to restore data parity (see section “2 Materials and methods”). Distinct behaviors occurring only in a dyadic context would have still been visible with this approach, like behavioral syllables related to self-grooming (see Supplementary Figures 4C, D). While some distinct behaviors (self-grooming and jumping) were captured as individual syllables as expected, these occurred too rarely to be included in our analysis (see section “2 Materials and methods”).

It was possible to detect contact-specific changes in the behavioral syllable composition for individual mice (Figure 4B, left panel), but we could not observe the same prominent effect for the joint syllable composition across two mice (Figure 4B, right panel). This difference in results between individual mice and two mice is very likely because joint occurrences of syllables are sparse, and a higher number of joint occurrences are necessary when analyzing the joint behavior of two mice. Since the significant effect of contact occurrence on the syllable distribution in individual mice is clear in our dataset, we decided to show the across-mice effect as a comparison to encourage further studies. Perhaps, an approach to decompose multi-animal behavior, from large datasets to overcome sparse social events, instead of decomposing individual animal behavior could be beneficial in detecting “social” syllables.

Social interactions capture rich and multi-modal behavioral repertoire (Porcelli et al., 2019) and thus need a data-driven extraction of behaviors. Recent studies addressed this need by using either parametric methods (Peleh et al., 2019a) or machine learning applications (Goodwin et al., 2024; Lin et al., 2024). It is also necessary to aim for explainable readouts from data-driven analysis of complex behaviors and a fruitful approach is to limit analysis to a small set of well-defined parametric approximations. We applied this perspective in our analysis (Figure 5C) and defined a set of four parametric categories in the context of mouse interaction (close contact, approach, leave, other stationary behaviors). Our original expectation with the behavioral decomposition approach was to find distinct syllables or sequences of syllables (syntaxes) that represent the parametrically defined categories. While we did find syntax families that capture most occurrences of social approach and leave (Figures 5C, D), a direct and distinct mapping of behavioral syllables onto social behavior classes was not observable (Figures 5E, G). It is possible that behavioral decomposition based on the key points may have contributed to the lack of distinct mapping of behavioral syllables onto social behaviors in our dataset because extraction of key points inherently limits the amount of pose information unlike pose extracted from depth recordings (Lin et al., 2024). In addition, a fruitful approach could be to use the joint pose information between mice, and this requires overcoming the limitation of sparseness in social interaction datasets.

In summary, our results show that, in the presence of a conspecific, mice possess a subset of behavioral syllables that were significantly modulated, and these modulated syllables change their transitory hub-like role in the transition network of all syllables. The presence of modulated syllables was not affected by the sex of the animals, the pause between recordings, the delivery route of the vehicle treatment, or the conspecific being limited to a cubicle. While it is to be expected that mice in the dyadic context are less stressed due to social stress buffering effects, we were also able to show that the number of DM syllables only grew when analyzing later segments of the solitary recordings. We did not find bidirectional mapping between a syllable or syntax and specific social behavior, yet our results show that syllables or syntaxes are sensitive descriptors to detect changes in behavior because of change in social context (Figure 6C). This finding taken together with previous studies, showing the sensitivity of behavioral syllables to detect different pharmacological interventions (Wiltschko et al., 2015, 2020), suggests that manipulation of the experimental context through social or non-social enrichment offers a valuable dimension for the phenotypic screening of drugs using behavioral decomposition methods.

## Data Availability

The datasets presented in this study can be found in online repositories. The names of the repository/repositories and accession number(s) can be found below: https://doi.org/10.5281/zenodo.15237564. The code used to generate the data shown in this study is publicly available. It can be found here: https://github.com/Marti-Ritter/social-context-restructures-behavioral-syntax-in-mice.
