# Supplementary material for: Social context restructures behavioral syntax in mice
Source: Front Behav Neurosci. 2025 Nov 7;19:1617091. doi: 10.3389/fnbeh.2025.1617091 (PMC12634660; doi:10.3389/fnbeh.2025.1617091)
Supplement: Supplementary file 1 [file Supplementary_file_1.pdf]

1 Supplementary data collection and analysis

1.1 Body center tracking

To explore any differences in mouse behavior, body center tracking was used to calculate the distance moved by a single mouse (Figure 2D), its position (Figure 5A), or inter-mice distance (Figure 5B-E, G). For tracking of body center, we used the mouse centroid calculated in Keypoint-MoSeq by taking the median of all used body points (10 points on the mouse body, excluding the tail). We then converted this pixel-based measure to a distance-based measure using the pixel per millimeter resolution in our arenas, calculated based on the known distance between the corners of the arena and the same distance in pixel measured in the video (measure extracted through the Python package OpenCV2, version 4.11.0; using the `selectROI` function).

1.2 Trajectory analysis

We extracted the syllable trajectories from the tracking information collected across all mice. To evaluate similarity between a pair of syllable trajectories, we calculated the cosine distance between position vectors of the pair of syllables, using the implementation in SciPy (version 1.14.0). Cosine distance is a measure of distance between two vectors (u and v) calculated as $\mathbf{d_{cos}} = \mathbf{1} - \frac{\mathbf{u} \cdot \mathbf{v}}{\|\mathbf{u}\|_2 \|\mathbf{v}\|_2}$. The cosine distance between all pairs is used for hierarchical clustering of syllables in a dendrogram (Figure 3A, Suppl. Figure 5A). The data shown in Figure 2 is recorded from the vehicle treated animals used in this study. As this study was part of a larger experiment, 40 additional mice of the same characteristics were recorded in parallel. When we used the larger dataset and looked at the hierarchical clustering of syllables in a dendrogram (Suppl. Figure 5A), we found 6 out of 8 dyadic-modulated syllables being separated into a distinct arm of the dendrogram.

The dendrograms shown here were created based on the output of the functions `linkage` and `dendrogram` contained in the SciPy package (Version 1.14.0) for Python. The exemplar trajectories for each syllable (Suppl. Figure 3A) were selected by the same algorithm as implemented in `get_typical_trajectories` in the Keypoint-MoSeq package for Python.

1.3 Eigenvector centrality

To identify syllable nodes that may play transitory role in a directed network of syllable transitions, we calculated eigenvector centrality measures for each syllable in the transition network of syllables in solitary and dyadic context (Figure 3B). Eigenvector centrality measure allows for the detection of nodes that are influential in the network, but not necessarily connected to many nodes themselves. The eigenvector centrality is defined by the formula:

$\mathbf{x_v} = \frac{1}{\lambda}\sum_{\mathbf{t}\in\mathbf{M(v)}} \mathbf{x_t}$, with $\mathbf{M(v)}$ being the set of neighbors of node $\mathbf{v}$. To calculate eigenvector centrality, we used the `eigenvector_centrality` function implemented in the NetworkX Python package (version 3.3) on the normalized transition probabilities (outgoing transitions sum to 1 for a given source syllable) for each animal in solitary and dyadic contexts. Overall, for a given syllable, we obtained 20 eigenvector centrality measures in each of solitary and dyadic contexts (Figure 3B).

1.4 Kullback-Leibler Divergence

To measure differences between distributions of syllables (Figure 4B) and syntaxes (Figure 5F), we applied the Kullback-Leibler divergence (D_{KL}), or relative entropy. The D_{KL} measures the distance between an observed probability distribution and an expected probability distribution. It is defined as $\mathbf{D_{KL}(P||Q)} = \sum_{\mathbf{x}\in\mathbf{X}} \mathbf{P(x)}\ \mathbf{log}\left(\frac{\mathbf{P(x)}}{\mathbf{Q(x)}}\right)$, with P being the observed and Q being the expected distribution. In this study we used a base of 2 for the logarithm, leading to a relative entropy in bits. To calculate the Kullback-Leibler divergence, we used the implementation contained in the `entropy` function in SciPy (passing the distribution to be compared as `pk` and the reference (expected) distribution as `qk`).

1.5 Parametric Behavior Classes

To gain a separation of behavioral syntaxes into parametrically defined behaviors, we used two parameters - inter-mouse distance (IMD) and change in distance (CID) as shown in Figure 5C. The peaks from kernel density plot in Figure 5C were separated into corresponding contact related class (syntaxes starting and/or ending below an IMD < 100mm) and control class (marked with hatched lines). Movement classes (approach and leave) were defined with an absolute CID > 50mm and a contact threshold of 100mm based on the preferred IMD shown in Figure 5B. The relative movement threshold (absolute CID) of 50mm was chosen as half of contact threshold. These two thresholds along with limitations of the arena (maximum IMD and maximum CID ≤ diagonal of arena) resulted in the polygons defining approach and leave behavior classes.

Control classes were defined as those that did not lead to inter-mouse contact but matched the same movement parameters (see dashed arrows in Figure 5C). For example, the control for approach behavior was any movement that had an CID < -50mm and led to a final IMD between 100mm and 200mm. The leave control class was defined as CID > 50mm and a final IMD between 100mm and 200mm. Finally, the contact control class used a larger distance criterion and captured the largest peak in the IMD-CID distribution, at an IMD > 200mm and an absolute CID < 50mm.

1.6 Hamming Distance

The hamming distance between two sequences is the number of positions that are different in the two sequences. This only includes substitutions, but not rearrangements. In this study, we defined the syntax family as a set of syntaxes with hamming distance < 1 relative to the name-giving syntax – for the syntax family (9,0,5), the syntax (9,0,10) would be a member but not (0,5,10). This was used to aggregate the diverse, but highly similar set of syntaxes influencing inter-mouse approaches and leaves as shown in Figure 5D. Further analysis of these families can be found in Figure 5E.

The formula for two sequences of identical length could be written as $d_{Hamming} = \sum_{i=1}^{n_x} eq(x_i, y_i)$, with x and y representing the two sequences, and n_x being the length of sequence x.

1.7 Principal Component Analysis (PCA)

We used dimensionality reduction with PCA to evaluate if dyadic-modulated (DM) syllables have a larger contribution to the variability across videos (keypoint tracks) of mice in solitary and dyadic contexts. We used syllable frame proportions for each individual mice, concatenated across animals, in solitary and dyadic contexts as input to the PCA. Since syllable frequencies summed up to 1, we did not apply further scaling to individual frequencies. The loadings for the first 15 PCs are split for DM or DU syllables (Figure 5A). Similarly, we also evaluated the contribution of DM syntaxes to the variability across videos (keypoint tracks) of mice in solitary and dyadic contexts (Figure 6B). We used the implementation of PCA in the scikit-learn package (version 1.5.1).

2 Statistical tests

All statistical tests were performed in Python (version 3.11.9). Unless stated otherwise we used a significance threshold of $\alpha=0.05$ and corrected for multiple testing by applying the Bonferroni correction where applicable.

2.1 Mann-Whitney U-Test

We used the Mann Whitney U-test in the following analysis:

- To verify that there were no light cycle modulated syllables, we applied a two-sided test.
- In Figure 2D, we tested if the total distance moved differed between solitary and dyadic recordings. Applying a one-sided test showed a significant difference between both groups ($p<0.0001$).
- To find DM syllables, we applied a two-sided test in Figure 2E.

- As mentioned in the section on the eigenvector centrality measure, we tested if eigenvector centrality differed between DM and DU syllables, as shown Figure 3B. We applied a two-sided test on the eigenvector centrality for DU and DM syllables, extracted per recording and compared between solitary and dyadic recordings. 5 out of 8 DM syllables showed a significantly different eigenvector centrality ($p \leq 0.05$) between solitary and dyadic recordings. No DU syllables showed a significant difference.
- Based on the results shown in Figure 3C, we tested for significantly modulated transitions between solitary and dyadic contexts. We applied a two-sided test to the transition counts observed in dyadic and solitary context for each transition observed in both contexts. After correction for multiple testing, we found 9 significantly modulated transitions with 8 of those targeting DM syllables.
- We also applied a one-sided test on the absolute value of the PCA loadings shown in Figure 6A, B to verify whether DM syllables or syntaxes provided a greater influence on the first 5 PCs compared to DU syllables or syntaxes. The tests showed that DM syllables had significantly higher loadings than DU syllables ($p < 0.002$), but that the same effect was not visible in DM syntaxes when compared to DU syntaxes.
- The data shown in Suppl. Figure 3B was tested with a two-sided test and we did not find significant differences between dyadic groups and the corresponding solitary onset proportions (after correction for multiple comparisons).
- For each parameter as shown in Suppl. Figure 3C, we performed a one-sided test between DM syllable trajectories and DU syllable trajectories and found the two direction-based parameters to be significantly reduced for the DM syllables (after correction for multiple comparisons).
- A two-sided test was applied for the pairwise comparisons of the data shown in Suppl. Figure 4B. We found that all the comparisons were significant (after correction for multiple comparisons).
- The comparison between the count of unique syllable transitions observed in dyadic context and solitary context yielded a significant result when we applied a one-sided test.
- The comparisons shown in Suppl. Figure 9 used a two-sided test.

We used SciPy's (version 1.14.0) implementation of the Mann-Whitney U-Test (function `mannwhitneyu`) with significance levels set at $\alpha=0.05$.

2.2 χ^2 Contingency Test

The χ^2 contingency test is used to test for independence of frequencies of two groups summarized in a contingency table. To verify which DM syllables showed a significant effect of experimenter-scored contact in Figure 2F (left panel), we applied this test to the onset counts of

each syllable during and outside contact compared to the onset counts of any other syllable during and outside contact. The test revealed two syllables (7 and 18) with significant effects ($p \leq 0.05$, Bonferroni correction). In Suppl. Figure 8A, we applied the χ^2 contingency test to the onset counts of each syllable family within or outside the behavior class, compared to the onset counts of all length 3 syntaxes within or outside the same behavior class. We found all comparisons to be significantly different, except for the comparison between syntax family (11,2,8) to all other syntaxes in the leave control behavior class. We used the implementation (`chi2_contingency` function) contained in the SciPy package for Python.

2.3 Two-way ANOVA

In Figure 2F, we compared the onset proportion of each DM syllable between solitary and dyadic contexts. Within dyadic context, we tested for an interaction between onset proportions of DM syllables and contact between conspecifics. Both contact (either experimenter-scored or based on distance quartiles; for distance distribution, see Figure 5B) and DM syllable were categorical variables. We performed a two-way ANOVA (formula `proportion ~ C(syllable) * C(contact)`) with the help of the `anova_lm` and `ols` functions contained in the statsmodels package (version 0.14.2) for Python. Results showed significant main effects on proportion and interactions between the categorical dependent variables (see Results).

2.4 Bonferroni correction

We corrected for multiple comparisons by using the Bonferroni correction. For example, we used the following equation to adapt the significance threshold for the 32 syllables to maintain our desired overall α=0.05: $\boldsymbol{\alpha}_{\mathbf{Bonferroni}} = \boldsymbol{\alpha}_{\mathbf{uncorrected}} / \mathbf{n}_{\mathbf{tests}}$ with $\mathbf{n}_{\mathbf{tests}}$ corresponding to the number of syllables. This correction was applied to the results of the χ^2 contingency test used in Figure 2F ($\mathbf{n}_{\mathbf{tests}}$=8); Mann Whitney U-tests in Figure 3B ($\mathbf{n}_{\mathbf{tests}}$ =32), Figure 3C ($\mathbf{n}_{\mathbf{tests}}$ =99), Suppl. Figure 3B ($\mathbf{n}_{\mathbf{tests}}$ =6), Suppl. Figure 3C ($\mathbf{n}_{\mathbf{tests}}$ =3), Suppl. Figure 4B ($\mathbf{n}_{\mathbf{tests}}$ =6); the χ^2 contingency test used in Suppl. Figure 8A **(**$\mathbf{n}_{\mathbf{tests}}$ =12).

2.5 Benjamini-Hochberg correction

To control for the false discovery rate in the Mann-Whitney U-test used to find the 8 DM syllables (see Figure 2C), the implementation of the Benjamini-Hochberg procedure provided by the SciPy package was applied. We used this in place of the Bonferroni correction as the visualizations provided in the Keypoint-MoSeq package also used the Benjamini-Hochberg correction. The same approach was applied to Suppl. Figure 9A-C. We also used this correction in the statistical test for light cycle modulated syllables.

2.6 Z-Score representation of syllable and syntax during contact

To identify whether the experimenter-scored contact behaviors had a significantly different representation in syllable space when compared to the global average of behavior, we

computed z-score for each of the syllable or syntax occurring during contact behaviors using the distribution of the same syllable or syntax in the predefined baseline window (Figure 4B, C). We calculated the z-score for data shown in Figure 4B and C using the following formula: $\mathbf{z} = \frac{\mathbf{x} - \mu}{\sigma}$.

In Figure 4B, a baseline period was defined between 5 seconds and 2 seconds prior to the scored contact. The z-score of each trace in Figure 4B was calculated based on the distribution of values for the same trace within the baseline window. In addition, we also calculated z-score for the active and passive contact traces based on the entire randomized trace (labeled "randomized" in the legend). We evaluated both z-scores framewise for every trace and defined frames as significant where both z-scores lay above 3. The frames found to be significant are marked for each trace in the upper subplot.

In Figure 4C, we again defined a baseline period between 5 seconds and 2 seconds prior to the scored contact. We computed z-score for each trace (syntax) based on the distribution of values for the same trace within the baseline window. We again defined a z-score above 3 as significant. All traces shown in black (active, n=25 and passive, n=24) showed significance across at least 50% of the test windows starting from 2 seconds prior to 2 seconds after the scored contact. The remaining traces are shown in gray (n_{active}=79, $n_{passive}$=80). Four syntax traces with the highest z-score were marked as the top 4 syntaxes associated with each contact type and are shown in color.
